# LHX6 is essential for the migration of human pluripotent stem cell-derived GABAergic interneurons

**DOI:** 10.1007/s13238-019-00686-6

**Published:** 2020-01-06

**Authors:** Fang Yuan, Kai-Heng Fang, Yuan Hong, Shi-Bo Xu, Min Xu, Yufeng Pan, Yan Liu

**Affiliations:** 1 Institute for Stem Cell and Neural Regeneration, School of Pharmacy, Nanjing Medical University, Nanjing, 211166 China; 2 Key Laboratory of Developmental Genes and Human Disease, Institution of Life Sciences, Southeast University, Nanjing, 2100096 China; 3 State Key Laboratory of Reproductive Medicine, Nanjing Medical University, Nanjing, 211166 China; 4 Co-innovation Center of Neuroregeneration, Nantong University, Nantong, 226019 China

**Dear Editor,**

GABAergic interneurons (GIs) play an essential inhibitory role in regulating neural circuitry and neurological activities. Approximately 54% GABAergic interneuron population originated from medial ganglionic eminence (MGE) at E13.5 in rodents (Butt et al., 2005), and migrated from MGE to dorsal cortex tangentially starting at E13–14 after the GABAergic neurons become postmitotic. Dysfunction of GABAergic interneurons causes severe neurological diseases, such as autism, schizophrenia and depression. Recent years, many studies have shown transplantation of GIs improved the neuronal function of animal models such as epilepsy, neuropathic pain, and fear erasure (Bráz et al., 2012; Cunningham et al.; Yang et al., 2016). Since migration is the intrinsic nature of GIs, regulation of GIs migration could be essential for cell therapy. However, the mechanism of human GIs migration remains unknown, which brings difficulties to control cell migration after transplantation.

According to the rodent studies, numerous transcriptional factors are involved in regulating GIs migration, such as Dlx1/2, Nkx2.1, and Lhx6. LIM Homeobox 6 (Lhx6) is a subtype gene of LIM homeodomain family. During early development, Lhx6 is expressed in the MGE and plays an essential role in the migration of GIs from MGE to the cortex in mice (Liodis et al., 2007; Zhao et al., 2008). Migration deficiency of GIs is found in *lhx6*-null mutant mice (Flandin et al., 2011). However, the role of the LHX6 in human GIs migration have not been investigated yet. Regulation of migration related transcriptional factors provide us a new prospective to promote GIs migration in cell therapy.

In order to study the role of LHX6 in modulating the migration of human GABAergic interneurons (GIs), we constructed LHX6 knockout hESC lines and differentiated into GIs. We constructed LHX6 knockout hPSC lines by using a donor-free paired gRNA-guided CRISPR/Cas9 strategy (Chen et al., 2015) (Fig. 1A). LHX6 knockout (KO) hESC colonies were used according to the results of DNA-sequencing (Fig. 1B). During GABA interneuron differentiation, the mRNA level of *LHX6* was dramatically decreased at day 17, and the expression of SHH was decreased in LHX6 KO cells whereas the mRNA levels of *NKX2.1* and *LHX8* did not change after knock out of *LHX6* (Fig. S1B), which is reasonable that cells in the MGE mantle area could enhance SHH signaling (Flandin et al., 2011). By day 22, the LHX6 KO hESC-derived GI progenitors showed similar expression of NKX2.1 compared to controls by using immunostaining, while an increased level of COUPTFII, a transcription factor of the caudal ganglionic eminence (CGE) (Fig. S1A and S1C). In addition, OLIG2, a subventricular zone marker of the MGE area, was significantly decreased in the LHX6 knockout group, while the FOXG1 was not altered (Fig. S1A and S1C).

**Figure 1:**
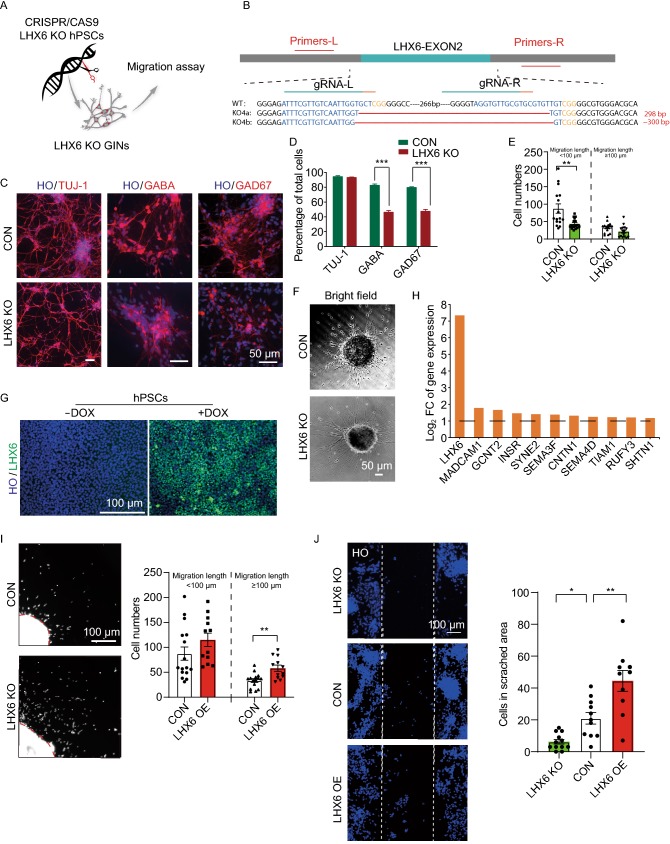
LHX6 regulated human GIs migration *in vitro*. (A) Schematic diagram of the analysis of GIs derived from LHX6 KO hPSCs. (B) Genomic DNA analysis for two LHX6 KO colonies. (C) Representative images of TUJ-1, GABA and GAD67 expressing in CON and LHX6 KO group. (D) Quantification of TUJ-1, GABA and GAD67 in CON and LHX6 KO group. (E and F) The LHX6 KO hESC-derived GIs showed decreased migration ability in an explant migration assay after attachment for 24 h. Scale bar, 50 μm. Quantification of migration in (F). At least 15 neurospheres were counted from each group, *n* ≥ 3 for each group. Bar graphs were presented as mean ± SEM. (G) Immunostaining of LHX6 in hPSCs after doxycycline treatment; (H) Fold change of migration related factors. (I) LHX6 OE hESC-derived GIs showed increased migration ability in an explant migration assay after attachment for 24 h. Quantification of migration at right panel. (J) Hoechst staining of hPSC-derived GIs in the control, LHX6 KO and LHX6 OE group in the scratch assay. The right panel is the quantification of the percentage of cells that migrated into the scratch area after 1 day post-attachment. Bar graphs were presented as mean ± SEM; *n* ≥ 3

By day 35, the percentage of GABA+ cells and GAD67+ cells in total cells was around 40% in KO, while around 80% in control group, indicating knockout of LHX6 decreased the GIs differentiation from hESCs (Fig. 1C and 1D). Strikingly, the explant experiment demonstrated that the LHX6 knockout GABA interneurons exhibited reduced migratory ability after plating for 24 h (Fig. 1E and 1F), indicating that LHX6 was crucial for the migration of GABA interneurons.

Since LHX6 KO suppressed GIs migration, we determined to further explore if LHX6 overexpression (OE) could promote GIs migration. LHX6 conditional overexpression human pluripotent stem cell (hPSCs) lines was constructed as our previous report (Yuan et al., 2018). GIs were robustly differentiated in both LHX6 OE and control group. SAG, an agonist of sonic hedgehog signaling pathway was used for ventral patterning (Fig. 1G). By 3 weeks of differentiation, over 80% of hPSC-derived cells expressed NKX2.1 (Fig. S2A and S2B), indicating the cell identity of medial ganglionic eminence (MGE) progenitors. By day 35, 90% of cells were TUJ1+ and 75% of cells were GABA+ (Fig. S2A and S2B) in both groups.

In retrospect of the RNA-seq profiles of LHX6 overexpression GIs (Yuan et al., 2018), we found migration-related GO terms were highly enriched in LHX6 OE GIs compared with control group (Fig. S2C). According to GO annotation of biological process, positive regulation of cell motility-related terms were enriched in LHX6 OE group. In addition, numerous genes associated with positive cell migration such as *SYNE2*, *SEMA4D*, *TIAM1* (Tanaka et al., 2004; Zhang et al., 2009; Kuzirian et al., 2013) were increased following *LHX6* overexpression (Fig. 1H). These results demonstrated that overexpression of LHX6 may positively promote GIs migration in gene profile.

To ascertain whether overexpression of LHX6 regulates migration of human GIs, we performed explant assay after attachment. On one day post-attachment, the LHX6 OE GIs showed a significant increasement in the number of cells that migrated more than 100 μm distance, in comparison with the control group (Fig. 1I). To further confirm the effect of LHX6 on cell migration *in vitro*, we cultured the GIs as single cells and performed scratch assay to determine the migratory ability. A few more cells migrated to scratched area in LHX6 compared with control group and fewer cells migrated in LHX6 KO group (Fig. 1J).

To further confirm that LHX6 regulates the migration in human GIs, we injected 5,000 7-week hPSC-derived GI progenitors onto the MGE area of embryonic day 15 (E15) mouse brain slices (Fig. 2A). After two weeks of co-culture, extensive ventral to dorsal migration was observed in LHX6 overexpression group in the direction of the MGE to the cortex. Human nuclei (hN) was used to label the grafted cells. We detected hN and GABA co-labeled cells in both groups. Notably, in similar areas of the brain slices, a dramatically increased number of hN cells were found in LHX6 overexpression group (Fig. 2B–D). Moreover, human GABAergic interneurons also showed an increased number in LHX6 OE group (Fig. S4). A distinct migration trace clearly showed that the grafted cells migrated from the injection site to the cerebral cortex in LHX6 overexpression transplants (Fig. 2E). To analyze the migration, the brain slices were divided into five areas based on the distance from the transplantation sites (Fig. 2F). At a distance of 0.75–2.25 mm, the number of migrated cells were similar in the two groups (+/− DOX). Significantly, more LHX6 overexpression GABA interneurons migrated into the area that was 2.25–3 mm away from the injection site (Fig. 2F and 2G).

**Figure 2:**
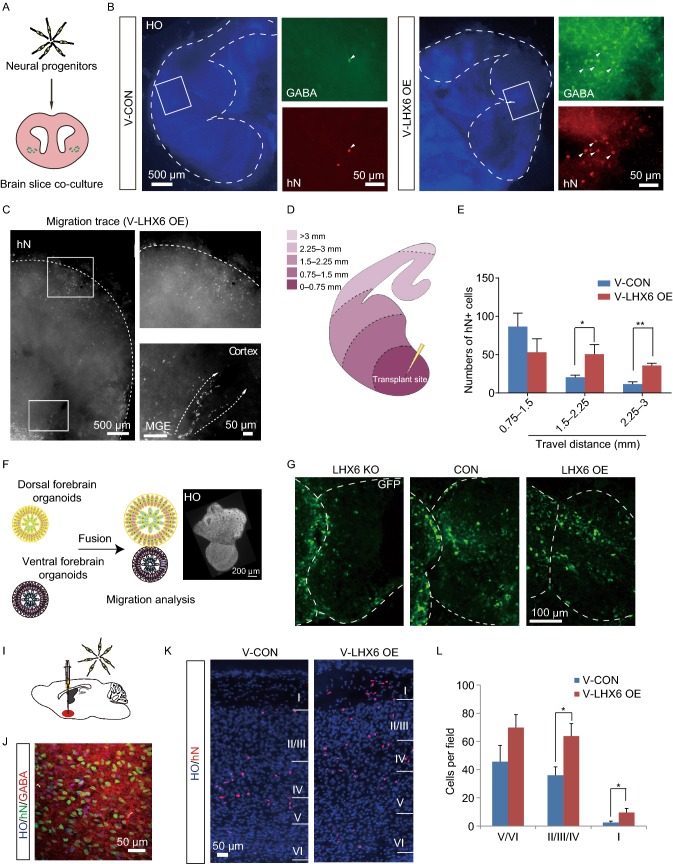
Human GIs migration was promoted by LHX6 OE in brain slice co-culture and *in vivo* transplantation. (A) Schematic representing mouse brain slices and the hPSC-derived GIs co-culture experiment. Human GIs were injected into the MGE area of E15 brain slices. (B–D) Mouse brain slices were co-cultured with control and LHX6 OE hPSC-derived GIs. The insets show similar areas of the brain slice, and the co-cultured slices were labeled with hN (human nuclei). (E) The migration trace of LHX6 OE cells. The white dashed arrow shows the migration trace of hN+ cells moving from the MGE to the cortex. (F) Schematic showing the division of the brain slices into different distances from the transplantation site. (G) Quantification of the numbers of hN+ cells at different distances in the co-cultured brain slices; *n* = 6 for V-CON and *n* = 5 for V-LHX6 OE. (H) Schematic diagram of fused forebrain organoids. Hoechst staining of fused organoids. Left side was a 35-day ventral forebrain organoid, right side was a 35-day dorsal forebrain organoid. (I) Cells labeled with GFP migrated from ventral organoids (left) to dorsal organoids (right). Quantification in (Fig. S3D). For each group, *n* ≥ 5. (J) Schematic showing the transplantation of hPSC-derived GABAergic progenitors into the basal forebrain of neonatal mice. (K) Immunostaining image of hN and GABA co-labeling. (L) Migrated hN+ cells in the cortical layers in the control and LHX6 OE groups. Scale bar, 50 μm. (M) Quantification of hN+ cells in each of the cortical layers in the control and LHX6 OE groups; *n* = 4 for V-CON and *n* = 5 for V-LHX6 OE

To more systematically test the role of LHX6 in migration, we used fused forebrain organoids to determine the GI migration from ventral to dorsal (Fig. 2H). Cerebral organoids and ventral MGE organoids were generated from hPSCs by following the reported protocols with slight modification, which mimic the endogenous developmental environment more closely than 2D culture (Birey et al., 2017) (Fig. S3A). To induce region-specific brain organoid, cerebral organoids were generated by default and ventral forebrain organoids were patterned with SAG, respectively. After 60 days culture from hPSCs, the cerebral organoids exhibited multiple cortical layer-like structures, with expressing cortical markers PAX6, TBR1, CTIP2, as well as neural progenitor markers SOX2 and NESTIN (Fig. S3B). 5 weeks old ventral forebrain organoids expressed NKX2.1, GABA and GAD67 (Fig. S3C). One week before fused, ventral organoids (OE, control and KO, respectively) were labeled with GFP by lentivirus infection, and we then fused the cerebral and ventral organoids in a well of 96-well plate for 3 days (Fig. 2I). As a result of this experiment, more GFP+ cells migrated from ventral organoids to dorsal organoids in LHX6 OE group (Fig. S3D).

In pioneering studies, transplantation of hPSCs derived GIs was reported to reduce seizure activities and held promise for neuropsychiatric diseases (Cunningham et al. 2014). To further investigate the migration of grafted human GABA interneurons *in vivo*, we transplanted the 7-week hPSCs-derived MGE progenitors into the ventral forebrain of severe combined immunodeficiency (SCID) neonatal mice (Fig. 2J). Both LHX6 OE group and control group were transplanted. After 3 months post-transplantation, most grafted cells differentiated into GIs that expressing GABA in human nuclei (hN) positive cells (Fig. 2K). Besides, a few grafted cells migrated from injected site (basal forebrain) to the dorsal cortex after 3 months (Fig. 2L). After transplantation, cells in LHX6 OE group were more likely migrated to superficial layers (Fig. 2M). Notably, the LHX6 overexpressed GIs showed increased migration ability and exhibited increased number of migrated cells in each layer of the cortex, whereas the control GIs showed fewer cells in the layer I–IV (Fig. 2L and 2M), indicating LHX6 overexpression significantly enhanced the cell migration to dorsal cortical layers *in vivo*.

Along with the technological advancement, GIs could be generated with high efficiency *in vitro*. Consequently, GIs transplantation has shown great therapeutic potential for many neurological disorders. Grafted cells might functionally integrate into host circuits and modify the activity of host cells after migration. However, very little was found in the regulation of human GABA interneurons migration after transplantation. Based on hPSC neural differentiation, we describe the role of LHX6 in human GABA interneuron migration by using knockout/overexpression strategies. In this study, we found that LHX6 is critical for controlling human GABAergic interneuron migration, which is confirmed with previous studies from *lhx6* transgenic rodents (Liodis et al., 2007). The GABAergic interneurons exhibited enhanced migration ability in cell culture, brain slice co-culture, cell transplantation and fused forebrain organoids upon overexpression of LHX6, while decreased migration ability with LHX6 knockout.

In our current study, LHX6 knockout cell line showed a decreased GIs differentiation efficiency (decreased GABA and GAD67 percentage), while in rodent studies that the GABA neuron number is similar in *lhx6* KO mice (Liodis et al., 2007). The percentage of COUPTFII positive cells increased in the LHX6 KO cell line, whereas decreased in the LHX6 overexpression cell lines in our previous observation (Yuan et al., 2018). Correspondingly, *lhx6* ko mice studies also showed Lhx6 could repress CGE-identity in MGE cells (Vogt et al., 2014). Therefore, our studies indicated LHX6 is important for the cell fate of ventral precursors and the generation of human GABA interneurons. We also used fused forebrain ventral-dorsal organoids to analyze human cortical interneuron migration, which is a comparatively ideal model to study human forebrain development. An interesting phenomenon was the size of dorsal organoids was vitally larger than ventral organoids (data not shown), suggesting the human cortical neural precursors may have stronger proliferative ability than ventral neural precursors. The result confirmed that LHX6 enhanced cell migration, and moreover, it applied a dynamic model for analyzing different human brain region interactions. Delayed migration of tangentially migrating GIs was found in Lhx6-deficient embryos (Liodis et al., 2007). Rodent studies showed that lhx6 could directly promote the expression of CXCR4/7 to form normal GIs migration (Vogt et al., 2014). However, in our RNA-seq data, CXCR4 showed no significant change and CXCR7 even downregulated in LHX6 OE group. It suggests human GIs migration might be regulated by a different and complex manner.

In conclusion, our results demonstrated that LHX6 is an essential transcription factor for the migration of human GABA interneurons by using knockout and overexpression strategies in human pluripotent stem cells. This method let us dig LHX6 function in human neurodevelopment from a new perspective. It could also be an important guide for the application of cell therapy in related diseases.

## Electronic supplementary material

Below is the link to the electronic supplementary material.

Supplementary material 1 (PDF 1016 kb)
