# Supplementary material for: LHX6 is essential for the migration of human pluripotent stem cell-derived GABAergic interneurons
Source: Protein Cell. 2020 Jan 6;11(4):286–91. doi: 10.1007/s13238-019-00686-6 (PMC7093371; doi:10.1007/s13238-019-00686-6)
Supplement: Supplementary file 1 — Supplementary material 1 (PDF 1016 kb) [file 13238_2019_686_MOESM1_ESM.pdf]

Supplementary materials

Materials and methods

Human pluripotent stem cell culture and neural differentiation

Human iPSCs (ihtc-03, Passage 10–20, established in our laboratory) were maintained with daily changed Essential 8 medium (Life Technologies) on vitronectin-coated plates (Life Technologies) at 37°C in a 5% CO2 incubator. Stem cells were passaged every 4-5 days by EDTA (Lonza) digestion. For neural differentiation, hPSCs were detached by 1U/ml dispase (Life Technologies) to form embryoid bodies (EBs) and then cultured in neural induction medium (NIM). After floating culture for 7 days, EBs were attached in a six-well plate with NIM supplemented with 5% FBS. Rosette structures could be observed in the center of attached colonies at day 10–16. At day 16, rosette colonies were carefully harvested manually with a 1 ml pipette to become neurospheres. Non-neuroepithelial clones were removed at this stage. Neurospheres were continuously floated in NIM to day 28 and then dissociated by TrypLE (Life Technologies) and plated on vitronectin (Life Technologies) and poly-l-ornithine (Sigma) pre-coated coverslips for further neuronal differentiation. 500nM SAG was added from day 10 to day 25 during differentiation. For induction of *LHX6* overexpression, DOX was added at 3 μg/mL since day 10.

Forebrain organoids culture and organoids fusion

For organoids culturing, embryoid bodies (EBs) were continuously cultured in neural induction medium (NIM) till day35 or longer time. For dorsal organoids patterning, hPSC derived organoids were cultured under spontaneous condition. For ventral organoids patterning, 500nM SAG was added from day 10 to day 25. Ventral organoids were infected with GFP lentivirus. To fuse dorsal and ventral organoids, one single dorsal organoid and one single ventral organoid were placed together in one 1.5ml EP tube at 37°C in a 5% CO2 incubator. After three days, fused organoids were fixed with 4% paraformaldehyde (PFA) for further analysis. For each group, n≥5. GFP+ Cells in ventral organoids were divided into two groups by the length from the soma location to the dorsal organoids' border line(<100 μm and ≥100μm, respectively). Cell numbers in this two groups were counted for quantification.

Human pluripotent stem cell electroporation

hPSCs were maintained under feeder-free conditions. hiPSCs or hESCs were treated with Rho Kinase (ROCK) inhibitor 24 hours before electroporation. Cells (1×10^6) were dissociated into single cells by dissociating with EDTA for 5 minutes and then were mixed with plasmids (OE: 20 μg donor plasmid and 5 μg TALEN arms; KO: 5 μg of a pair of gRNA plasmids, 5 μg Cas9 plasmid, and 5 μg PGK promoter-driven plasmids) using the Lonza Nucleofectorkit (VPH-5002). The cell mixture was electroporated in a Lonza Nucleofector 2b with the A027 program. After electroporation, the cells were quickly reseeded on a vitronectin-coated six-well plate in Essential 8 medium with ROCK inhibitor added for the first 24 hours. Stable colonies were selected after 5–7 days of continuous puromycin/G418 treatment.

Immunostaining for cells, organoid slices and brain slices

Cells cultured on coverslips were fixed in fresh 4% PFA for 30 min at room temperature and rinsed three times with phosphate buffered saline(PBS). Cells were treated with 0.2% Triton X-100-containing PBS for 10 minutes and blocked in 10% donkey serum for 1 hour (organoid slices/brain slices: 0.5% TritonX-100 and 5% donkey serum for 1 hour). Cells/ organoid slices/brain slices were incubated at 4 °C overnight in primary antibody diluted with 0.1% triton and 5% donkey serum. On the next day, cells/ organoid slices/brain slices were incubated in secondary antibody diluted in 5% donkey serum for 30 min at room temperature after rinsed three times with PBS. Samples were mounted for fluorescent imaging solution with Floromount-G(Southern Biotech). The primary and secondary antibodies were listed in Supplementary Table 1.

Quantitative real-time PCR

As previously described (Y. Liu, Weick, et al., 2013), total RNA was extracted in Trizol reagent (Invitrogen), and furthermore, cDNA was reverse-transcribed by using the SuperScript III First-Strand kit (Invitrogen). In addition, RT-PCR was performed using the Bio-Rad MyiQ real-time PCR detection system. Primers used were listed in Supplementary Table 2.

Scratch assay and explant migration assay

For the scratch assay, the progenitors were dissociated into single cells and planted on poly-l-ornithine/Matrigel-coated 96-well plates (10^5 cells/well) as previously described (Fattahi et al., 2016). After 24 hours of attachment, cells were scratched with a pipette tip in the middle of the well. Migrated cells were counted in the scratch area after 24 hours.

For the explant migration assay, the neurospheres (with a mean diameter of around 150 μm) were planted on poly-l-ornithine/Matrigel-coated 96-well plates (8–15 explants) without dissociation as described previously (Lin-Hendel, McManus, Wallace, Anderson, & Golden, 2016). After 24 hours, migrated cells from the neurospheres were divided into two groups by the migrated length (<100 μm and ≥100μm, respectively). Cell numbers in this two groups were counted for quantification.

Preparation of coronal telencephalic slices and co-culture with hPSC-derived MGE-like progenitors

Embryonic day 15 mouse telencephalic slices were sectioned with a McIlwain Tissue Chopper (MTC/2E) at a thickness of 250 μm. We selected the slices that contained separated lateral ventricles for further culturing on transwell membranes (Chemicon & Millipore). Embryonic brain slices were maintained in the medium containing B27 (Life Technologies) and penicillin (Life Technologies). After culturing embryonic brain slices for 24 hours, MGE area of the telencephalic slice was marked by objective marker. To prepare the transplantation, hPSC-derived progenitors were broken into little clusters with diameters of around 30 μm 2–3 days previously. 8,000 day-35 neural progenitors were transplanted

into the circled area by tips. Brain slices and human grafted cells were co-cultured for another two weeks for further migration assay.

Animals and neonatal transplantation

Model Animal Research Center of Nanjing University provided the SCID mice used in our experiment. After approved by the Animal Care and Use Committee at Nanjing Medical University, all of the animal experiments were performed strictly with standard experimental protocols. The postnatal day (P0) SCID pups were randomly divided into two groups and were injected with 1 μl day-35 V-CON/V-*LHX6* overexpression MGE-like progenitors at a density of 1.5×10^5 cells/μl. To prepare the transplantation, hPSC-derived progenitors were broken into little clusters with diameters of around 30 μm 2–3 days previously. On the day of transplantation, cells were suspended in 10–20 μl NIM with B27 (Life Technologies) and penicillin (Life Technologies). Both hemispheres of each neonatal pup were injected with 1μl cell suspension using a glass micropipette. The injection site was in the basal forebrain. Finally, the pups were laid on a pre-warmed cushion for 15 minutes and then returned to their cage after injection.

Cell counting and statistical analysis

Images were captured using an Eclipse 80i fluorescence microscope and ZEISS LSM 700B confocal microscope. The quantification of fluorescent images was completed in the platform of Image J. More than 1,500 cells for each index were counted in each cell line, and at least three duplications were performed in each experiment. The total cell number was achieved by counting the number of nuclei marked by Hoechst *in vitro* experiments, and the number of nuclei labeled by hN were referred to the total grafted cell numbers in the transplantation experiment. More than 2,000 hN+ cells were counted in each index, n (V-CON) = 4, n (V- *LHX6* overexpression) = 5).

Student's t-test, one-way ANOVA, and two-way ANOVA were utilized during data analysis. All graphical data were presented as mean ± SEM while values were considered statistically significantly different at $p < 0.05$(*), $p < 0.01$(**), and $p < 0.001$(***).

Supplementary figures

Figure S1

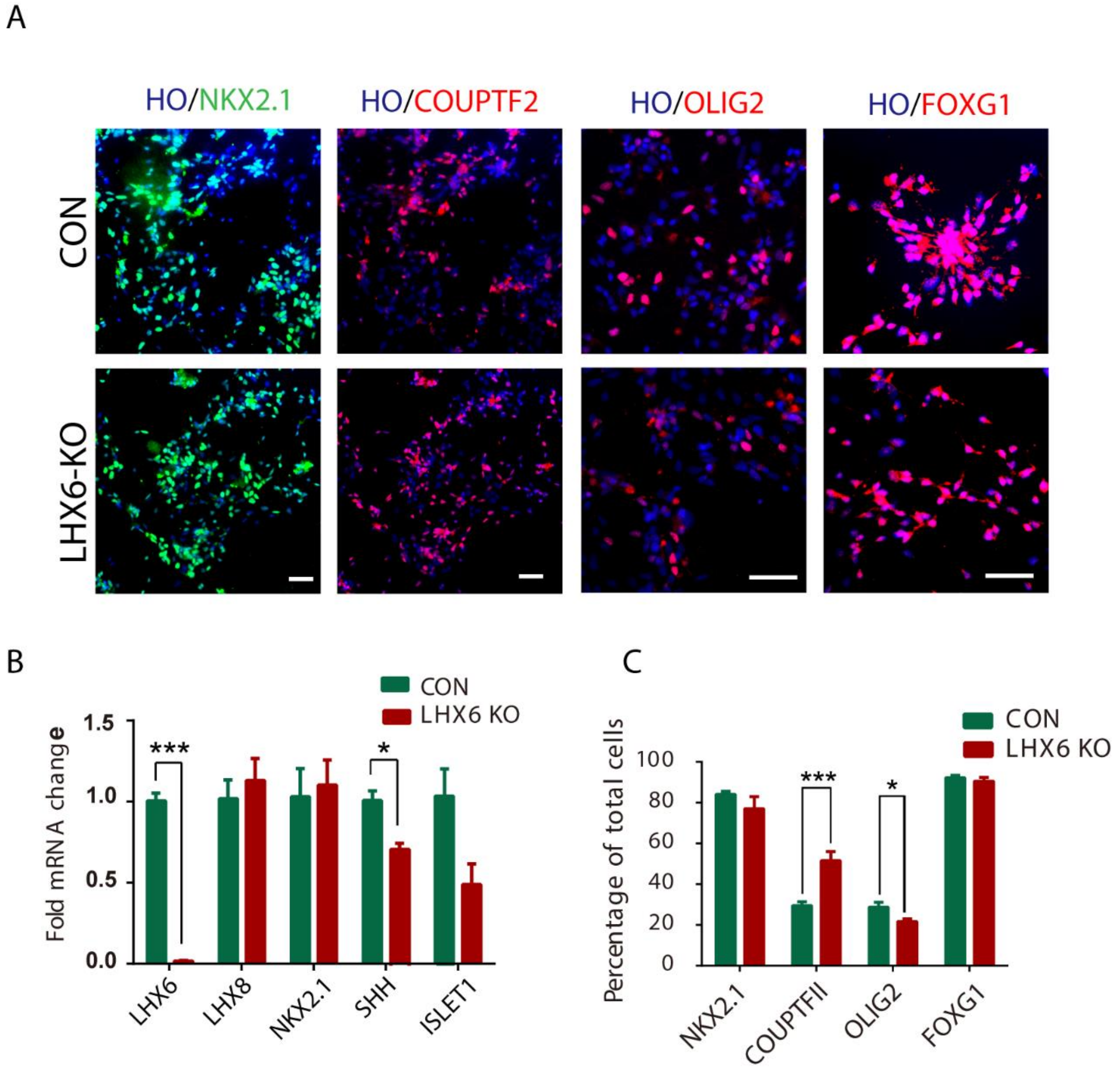

Figure S1

A. Representative images of NKX2.1, COUPTFII, OLIG2 and FOXG1 expressing in CON and LHX6 KO group. Scale bar, 50μm.

B. mRNA levels for control and LHX6 KO hESC-derived neurospheres at day 17; n≥3 biological replicates.

C. Quantification of NKX2.1, COUPTFII, OLIG2 and FOXG1 postive cell numbers in CON and LHX6 KO group.

Figure S2

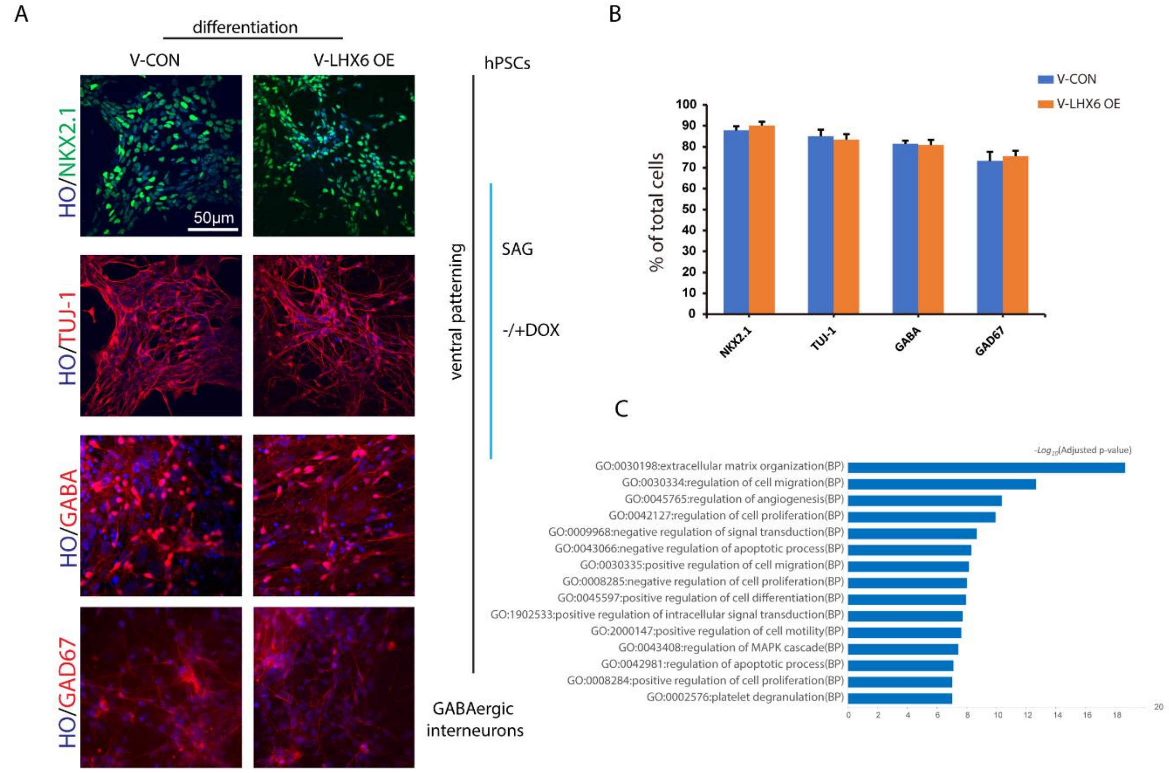

Figure S2

A&B. Representative images(A) and quantification(B) of V-CON&V-LHX6 OE groups expressing NKX2.1, TUJ-1, GABA and GAD67.

C. GO analysis of gene profiles from V-CON&V-LHX6 OE groups showed related terms .

Figure S3

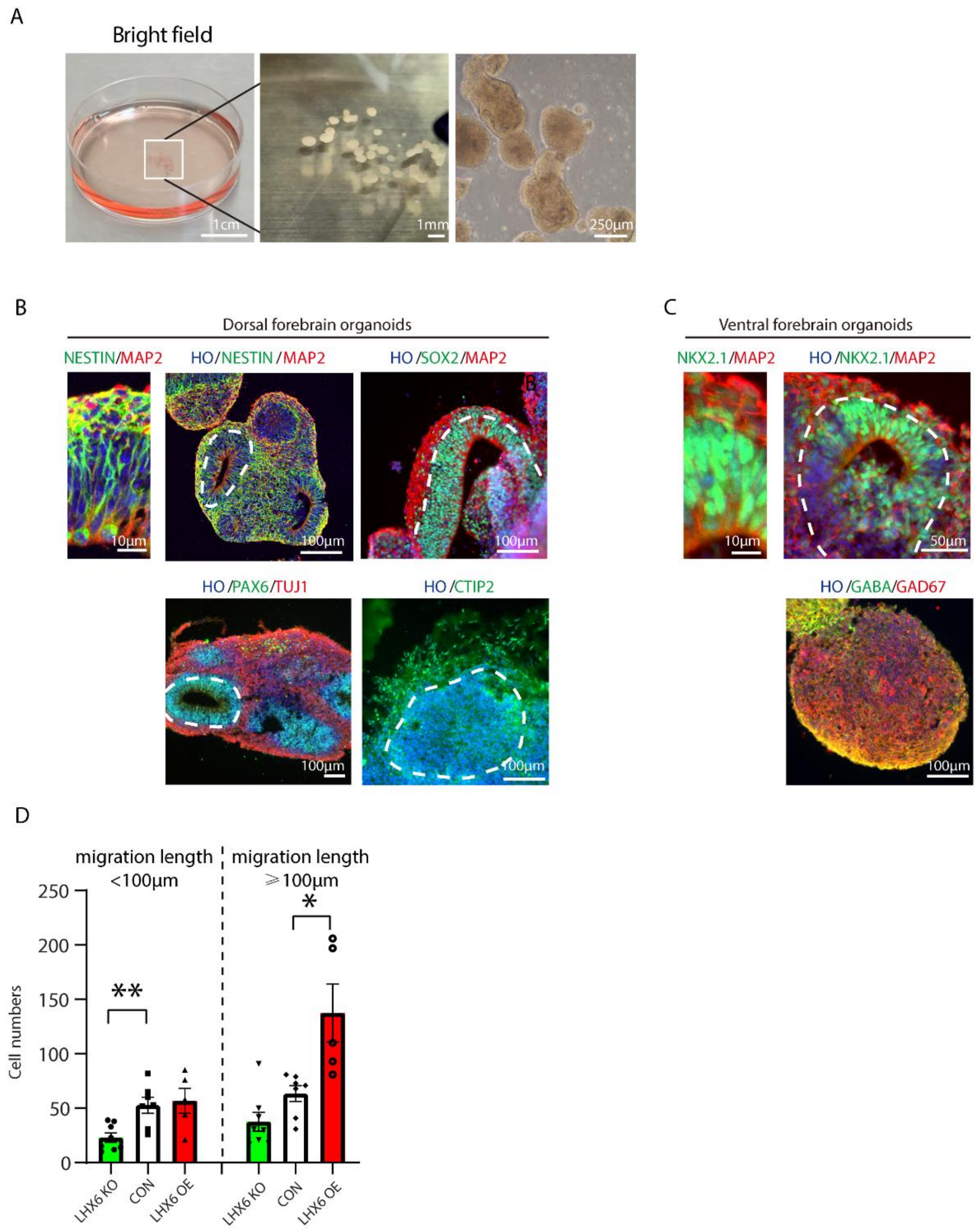

Figure S3

A. Bright field of organoids in suspension culturing.

B. Identification of dorsal forebrain organoids. Day 35 dorsal organoids expressed Nestin, SOX2, PAX6 , CTIP2 and MAP2.

C. Identification of ventral forebrain organoids. Day 35 ventral organoids expressed NKX2.1, GABA, GAD67 and MAP2.

D. Quantification of GFP+ cells migrated from dorsal organoids to ventral organoids.

Fig S4

A

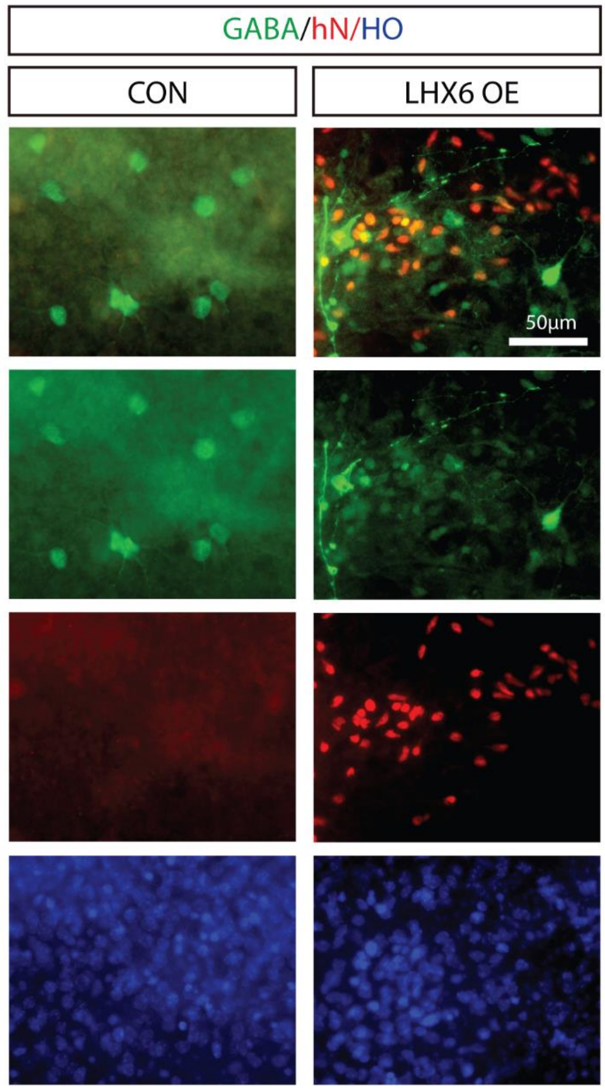

Figure S4

A. Reprehensive images of hN/GABA of mouse brain slices co-cultured with control and LHX6 OE cells.

Table S1

Genes related to Fig 1H.

Unigene	Length	V-CON-Ex	V-LHX6-GOF-Express		log2FoldC	Pvalue	Padj	Up/Down-	Symbol	Description
26468	3408	30.49	7963.96	1	7.3380293	0	0	Up	LHX6	LIM homeobox 6
8174	1431	2.42	15.72	1	1.7851815	0.0066841	0.0187466	Up	MADCAM	mucosal vascular addressin cell adhesion molecule 1
2651	4509	23.98	118.26	1	1.6599210	2.88E-10	1.95E-09	Up	GCNT2	glucosaminyl (N-acetyl) transferase 2, I-branching enzyme (I blood group)
3643	9339	85.71	365.51	1	1.4630093	1.62E-22	2.12E-21	Up	INSR	insulin receptor
23224	12056	326.37	1329.85	1	1.4062271	5.76E-65	2.23E-63	Up	SYNE2	spectrin repeat containing, nuclear envelope 2
6405	3401	69.29	278.39	1	1.3803554	2.24E-16	2.21E-15	Up	SEMA3F	semaphorin 3F
1272	5476	53.44	206.2	1	1.3141190	1.06E-11	7.98E-11	Up	CNTN1	contactin 1
10507	4569	85.72	314.66	1	1.2475632	6.53E-16	6.28E-15	Up	SEMA4D	semaphorin 4D
7074	7218	114.9	414.36	1	1.2263813	2.68E-18	2.91E-17	Up	TIAM1	T-cell lymphoma invasion and metastasis 1
22902	2995	345.7	1230.38	1	1.2145262	2.50E-36	5.19E-35	Up	RUFY3	RUN and FYVE domain containing 3
57698	4109	177.34	619.69	1	1.1778001	1.53E-22	2.01E-21	Up	SHTN1	shootin 1
650	3191	16.24	56.51	1	1.1283034	0.0014188	0.0045874	Up	BMP2	bone morphogenetic protein 2
